# Factors associated with bystander intervention in cyberbullying: a systematic review

**DOI:** 10.3389/fpsyg.2026.1912279

**Published:** 2026-09-11

**Authors:** Zhengmeng Wang, Xinxin Wei, Suriati Saad, Feroz De Costa, Tinglei Liu

**Affiliations:** 1 School of Communication, Universiti Sains Malaysia, Penang, Malaysia; 2 Universiti Putra Malaysia, Serdang, Malaysia; 3 Kuala Lumpur University of Science and Technology (KLUST), Kuala Lumpur, Malaysia; 4 Hebei Normal University for Nationalities, Chengde, China

**Keywords:** adolescents, bystander intervention, cyberbullying, cyberbystander, defending behavior, MMAT, PRISMA, systematic review

## Abstract

**Introduction:**

Cyberbullying among adolescents often takes place in front of an online audience, yet many bystanders remain passive. Evidence on factors associated with intervention is dispersed across psychological, relational, contextual, and platform-related areas.

**Methods:**

Following PRISMA 2020 and PRISMA-S, we searched Web of Science Core Collection, Scopus, ScienceDirect, SpringerLink, PubMed, and IEEE Xplore, together with citation searching and Google Scholar, for empirical studies published from 2018 to June 2026. Database searches identified 1,244 records and yielded 41 included reports; citation searching and Google Scholar identified eight additional reports, resulting in 49 reports overall. Conclusions about adolescent intervention were anchored in a 42-report core corpus, with seven reports at the population, phenomenon, or outcome boundaries examined separately. Included reports were appraised using the Mixed Methods Appraisal Tool (2018 version) and synthesized through a structured narrative approach across six a priori factor domains without meta-analysis.

**Results:**

The most consistent evidence concerned individual psychological resources, especially empathy, defender self-efficacy, and constructive moral orientation. Incident appraisal and relational, family, and school ecology were linked to intervention more conditionally, while demographic, sociocultural-normative, and platform-design factors were mixed or sparse. Most reports used cross-sectional designs and self-report measures, and the evidence was concentrated in a limited number of national settings.

**Discussion:**

The findings can be organized in a staged ecological framework moving from recognition and incident appraisal to moral-emotional activation, efficacy and norm calibration, and action selection. They may inform empathy and efficacy training, safe reporting routes, and supportive school, family, and peer conditions in adolescent school settings similar to those represented in the evidence; platform-level and cross-cultural applications require local adaptation and direct testing.

**Systematic review registration:**

https://doi.org/10.17605/OSF.IO/68CMH, identifier: OSF.IO/68CMH.

## 1 Introduction

Cyberbullying is conventionally defined as aggression carried out repeatedly through electronic contact against a target who cannot easily defend themselves (Smith et al., 2008). Compared with offline bullying, it is intensified by anonymity, persistence, and networked visibility. Anonymity lowers the perceived cost of aggression, humiliating material can be archived and re-encountered long after the original incident (Karthikeyan, 2022), and a single hostile post reaches a large audience within minutes. Aggression is therefore enacted in front of a spectating crowd, making cyberbullying a public and socially distributed event, and the problem now extends well beyond high-connectivity settings into rapidly digitizing societies (Nwufo and Nwoke, 2018). These adverse associations are supported by quantitative syntheses: a meta-analysis of 57 studies from 17 countries found a positive association between adolescent cybervictimization and depression [*r* = 0.291, 95% CI (0.246, 0.335)] (Hu et al., 2021), while a systematic review of 26 independent studies reported that cybervictimized young people had higher odds of self-harm, suicide attempts, and suicidal ideation than non-victims (John et al., 2018). These observational syntheses do not establish that cyberbullying alone causes the outcomes, but they establish a clinically consequential association. The search for scalable points of intervention has therefore turned beyond victims and perpetrators to the people who witness these incidents.

Bystanders occupy a consequential position because the trajectory of an incident depends substantially on how its witnesses respond. Classic work established that the presence of others does not guarantee help and may suppress it, as responsibility diffuses across a crowd (Latané and Darley, 1970). Online settings amplify these dynamics: audiences are larger and less identifiable, and most witnesses remain passive (Machackova et al., 2018). Passivity is consequential rather than neutral. Defending can interrupt the aggression and validate the victim, whereas silence can be read as tacit endorsement that emboldens the perpetrator (Song and Oh, 2018). Bystander responses also span a spectrum, from active defending through emotional or instrumental support and reporting to passivity and, at the negative extreme, reinforcing the aggression (Olenik-Shemesh et al., 2017). Treating these as distinct outcomes matters because the conditions that move a witness toward support need not be those that produce confrontation or formal reporting.

Despite a decade of attention, this literature remains fragmented in ways that limit its usefulness. Much work has documented non-intervention, leaving the question of what activates defending under-theorized (Song and Oh, 2018). Facilitators have typically been studied one factor at a time, within single cultural or platform settings, and with heterogeneous cross-sectional, self-report, and vignette designs. As a result, associations that appear robust in one population often fail to replicate cleanly in another. The accumulated single-factor findings have not been consolidated into an account of how these influences relate to one another, distribute across the levels of a young person's social ecology, or combine to move a witness from exposure to action. A systematic review can bring this heterogeneous evidence together, identify where findings converge, and examine the methodological sources of disagreement.

Several prior syntheses have begun this consolidating work, but each is bounded in scope, period, or framing (Table 1). The systematic review by Domínguez-Hernández et al. (2018) assembled 19 studies published between 2005 and 2016; its search predated the platform-era surge, and it offered neither formal quality appraisal nor coding of cross-cultural or platform-level influences. Polanco-Levicán and Salvo-Garrido (2021) mapped the literature published between 2015 and 2020, but on a base of nine articles and with an emphasis on classifying bystander roles rather than determinants. Rudnicki et al. (2023) reviewed bystander intervention in online hate and cyberbullying among adults, leaving the adolescent, school, family, and platform ecology largely unaddressed. Lo Cricchio et al. (2020) synthesized moral disengagement across all involvement roles rather than the broader factor set shaping intervention. Most recently, Jeyagobi et al. (2022) reviewed the factors behind negative cyber-bystander behavior (passivity, reinforcing, and aggressive defending) in studies published between 2012 and 2022, explicitly setting aside constructive intervention and without a formal quality appraisal. The present review instead asks what is associated with defending and support across the full bystander-response spectrum. Existing reviews identify important pieces of the problem, but they do not yet explain how these influences connect across the path from noticing to acting. This review updates and broadens that work. It restricts inclusion to studies published from 2018 to June 2026, searched across six databases plus citation searching, and synthesizes 49 studies, including a substantial body of recent work from Chinese samples that earlier reviews could not capture. Every included study is appraised with the Mixed Methods Appraisal Tool, and the factors are organized into a staged, multilevel account of intervention readiness.

**Table 1:** Prior systematic reviews of cyberbullying bystander behavior and the added value of the present review.

Review (author, year, venue)	Search period	Databases	Population (*n* included)	Main focus and appraisal	Added value of present review
Domínguez-Hernández et al. (2018), (Cyberpsychology: Journal of Psychosocial Research on Cyberspace)	2005–2016	Web of Science Core collection + SciELO citation index	Cyberbullying bystanders under 20 years of age (*n* = 19)	Systematic review of psychological and contextual moderators of bystander action (intervention vs. passivity) in cyberbullying. Appraisal: not reported	Updates to 2026 across six databases; 49 studies; MMAT appraisal; mapping of factor domains to process stages
Polanco-Levicán and Salvo-Garrido (2021), (Frontiers in Psychology)	2015–2020	Web of Science, PubMed, Scopus	Adolescent students; cyberbystander roles (*n* = 9)	Mini-review of cyberbystander roles and types (who, how many, and why) among adolescent students. Appraisal: not reported (mini-review)	Moves from roles to determinants and stages; 49 studies; PRISMA 2020 reporting; MMAT appraisal
Rudnicki et al. (2023), (Behavior & Information Technology)	NR	NR	Adults; bystanders of online hate and cyberbullying	Review of bystander intervention in online hate and cyberbullying among adults, conducted following the Cochrane Collaboration Handbook. Appraisal: Per the Cochrane Collaboration Handbook (tool not reported)	Supplies the youth-focused, cyberbullying-specific, multilevel synthesis
Lo Cricchio et al. (2020), (European Journal of Developmental Psychology)	NR	NR	Children and adolescents; cyberbullying involvement (perpetration plus active and passive bystanding, not bystander-specific) (*n* = 41)	Systematic review of moral disengagement and cyberbullying involvement (perpetration and active/passive bystanding). Appraisal: NR	Embeds moral disengagement in a six-domain, staged ecological model
Jeyagobi et al. (2022), (Frontiers in Public Health)	2012–2022	Scopus, ScienceDirect, Web of Science, SpringerLink, SAGE Journals	Children, adolescents and some adult cyber-bystanders; negative-behavior focus (*n* = 27)	Systematic review of factors driving negative cyber-bystander behavior (passivity, reinforcing, assisting, aggressive defending); excludes positive/constructive bystanding; grouped into personal, situational, and social-influence factors. Appraisal: not reported	Complementary positive-intervention focus across the full defending-to-reinforcing spectrum; MMAT appraisal of all 49 studies; staged ecological-process framework; coverage extended to June 2026 (adds PubMed and IEEE Xplore)
This review (2018–2026)	2018–2026	Web of Science, Scopus, PubMed, ScienceDirect, SpringerLink, IEEE Xplore + citation searching/Google Scholar (six databases + other methods)	Adolescent, youth, and student bystanders; 42 core + seven boundary reports	Factors associated with cyberbystander intervention synthesized into a multi-stage ecological-process framework (recognition, appraisal, moral-emotional activation, efficacy/norm calibration, action). Appraisal: MMAT (Mixed Methods Appraisal Tool)	NA (this is the current review)

NR, not reported.

Two frameworks motivate this staged, multilevel reading. The bystander-intervention decision model conceptualizes helping as a sequence of steps: the witness must notice the event, interpret it as requiring help, assume responsibility, decide how to help, and act, and failure at any step terminates the path to action (Latané and Darley, 1970). The logic fits cyberbullying, where ambiguous cues complicate interpretation, large invisible audiences dilute responsibility, and uncertainty about safe responses can stall a motivated witness. The socio-ecological systems perspective situates behavior within nested environmental layers, from family, peers, and school to community institutions and cultural norms (Bronfenbrenner, 1979). Together, these frameworks imply that the documented determinants act at different points in a decision sequence and at different levels of the social ecology, rather than as an undifferentiated list of parallel predictors.

This systematic review aims to identify, organize, and integrate the factors associated with bystander intervention in cyberbullying among adolescents and young people. It is guided by three research questions. RQ1: Which individual, situational, relational, sociocultural, and platform-related factors have been examined in the 2018–2026 literature, and how is each associated with bystander intervention? RQ2: How do these factors cluster when organized by factor domain and by position along the bystander outcome spectrum of defending, support, reporting, passive non-intervention, and reinforcing? RQ3: How can the clustered factors be organized into a process framework of intervention readiness that links exposure and recognition, incident appraisal, moral-emotional activation, efficacy and norm calibration, and action selection? Section 2 details the methods, Section 3 reports the synthesis, Section 4 develops the integrative model and its implications, and Section 5 concludes. To our knowledge, prior reviews have not jointly combined quality-appraised coverage of the positive, intervention-oriented end of cyberbystander behavior with an explicit mapping of individual, relational, sociocultural, and platform-level factors onto a staged process of intervention readiness. This review contributes to educational psychology by clarifying how individual, relational, contextual, and platform-level factors shape adolescents' bystander responses in cyberbullying, and by translating that synthesis into guidance for school-based prevention and for building supportive peer, family, and classroom environments.

## 2 Materials and methods

### 2.1 Protocol and reporting

This systematic review was conducted and reported in accordance with the PRISMA 2020 statement (Page et al., 2021), with the search documented following the PRISMA-S extension (Rethlefsen et al., 2021). The review protocol and transparency materials were archived on the Open Science Framework: https://doi.org/10.17605/OSF.IO/68CMH. The eligibility criteria, information sources, data items, and synthesis approach were documented and applied consistently during the final screening, verification, and synthesis stages. To improve transparency, the full search log, screening register, data-extraction sheet, MMAT appraisal workbook, and PRISMA 2020 and PRISMA-S checklists are available in the associated Open Science Framework project (https://osf.io/89s3r). Because the included studies differed substantially in design, in the operationalization of bystander behavior, and in the predictors examined, the evidence was not amenable to statistical pooling, and we adopted a structured narrative approach guided by the Synthesis Without Meta-analysis (SWiM) guideline (Campbell et al., 2020), as developed in Section 2.8.

### 2.2 Information sources

We searched six bibliographic databases spanning the psychological, social-science, biomedical, and computing literatures: Web of Science Core Collection, Scopus, ScienceDirect, SpringerLink, PubMed, and IEEE Xplore. The combination balanced multidisciplinary coverage, publisher-level depth in journals carrying adolescent online-behavior research, biomedical indexing, and the engineering venues in which platform-design studies appear. To reduce the risk that database indexing alone would omit relevant work, we added backward and forward citation searching of included studies and supplementary Google Scholar searches. All database searches were executed in June 2026, with eligibility restricted to records published from 2018 to June 2026; the 2018 bound concentrates the synthesis on the period in which bystander research moved beyond single-factor empathy accounts toward multi-determinant models. The per-database audit trail (interface, date searched, records retrieved) is reported in Table 2, and the full query strings and applied filters are provided in Supplementary Table S1.

**Table 2:** Database search strategy and PRISMA audit trail.

Database	Interface	Records identified	Role in PRISMA
Web of Science Core Collection	Clarivate Web of Science (Smart Search)	238	Databases/registers identification arm
ScienceDirect	Elsevier ScienceDirect (web search, show = 100 offsets)	473	Databases/registers identification arm
SpringerLink	Springer Nature SpringerLink (CSV download)	362	Databases/registers identification arm
Scopus	Elsevier Scopus (Article title/Abstract/Keywords)	106	Databases/registers identification arm
PubMed	NCBI PubMed (E-utilities esearch/efetch XML-to-CSV)	64	Databases/registers identification arm
IEEE Xplore, original	IEEE Xplore; natural-language query as executed	1	Databases/registers identification arm (8 June 2026)
All databases combined	Merge + deduplication (DOI/title)	1,244	Databases arm: 1,244 identified → 226 duplicates → 1,018 screened
IEEE Xplore, verification	IEEE Xplore; broader Boolean verification query	31 total; six journal; 0 eligible	Post-review verification (30 July 2026); not added to original PRISMA counts
Other methods	Google Scholar + citation/reference-list searching	Eight included; total candidates screened NR	Inclusion-stage count; an eight-of-eight retention rate is not claimed

Original database searches were conducted on 8 June 2026. The IEEE verification search was conducted on 30 July 2026. Exact original and verification strings are provided in Supplementary Table S1. NR, not recorded.

### 2.3 Search strategy

The original searches used the natural-language concept phrase “cyberbullying bystander intervention adolescents,” with platform-specific field or URL syntax where available. Supplementary Table S1 reproduces each query exactly as executed; we do not retrospectively recast those phrases as a more elaborate Boolean strategy. Searches were limited to peer-reviewed, English-language records; the language restriction is acknowledged as a potential source of bias and revisited in the Discussion. In response to the concern raised about the single IEEE Xplore record, we also ran a broader, explicitly separate verification search on 30 July 2026 using [“cyberbullying” AND (bystander OR witness OR defender)]. It returned 31 records, including six journal articles. Title-and-abstract screening of those six journal records found that all concerned computational detection, dataset construction, or automated role identification rather than empirical determinants of human bystander intervention, so the verification search added no eligible report. This post-review verification does not replace or alter the June 2026 search; it documents the sensitivity of the IEEE yield to query breadth. Table 2 and Supplementary Table S1 distinguish the original and verification searches.

### 2.4 Eligibility criteria

Eligibility was defined using a PICOS-style framework, summarized in Table 3. The primary population of interest was adolescents, youth, or students occupying a bystander or witness role in relation to cyberbullying directed at a peer; workplace samples and studies of general adult online behavior were outside that primary scope. For the phenomenon of interest, we included studies examining factors associated with how bystanders respond to cyberbullying online, and excluded studies addressing only perpetration or victimization. Eligible reports were empirical, peer-reviewed, in English, and published between 2018 and June 2026; work concerned solely with online hate speech or offline bullying was excluded. Quantitative, qualitative, mixed-methods, experimental, randomized, and psychometric designs were eligible because the determinants literature spans heterogeneous methods; reviews, theses, and preprints were excluded as document types. During synthesis, seven reports were found to sit at the population, phenomenon, or outcome boundary: the adult-sample reports by Wang and Kim (2021) and You and Lee (2019), the social-media-incivility experiments by Guo et al. (2025), the witnessing-distress reports by Doumas and Midgett (2020), Fabris et al. (2022), and Wright and Wachs (2022), and the psychometric validation by Alcántar-Nieblas et al. (2024). To avoid allowing these records to broaden the adolescent inference, we retained them for transparent mapping but did not use them as primary support for youth-focused conclusions. The corpus is therefore read in two layers: a 42-report core corpus and a seven-report boundary set. Section 3.4 reports the core-only and evidence-unit sensitivity analyses explicitly.

**Table 3:** Eligibility criteria and full-text exclusion codebook.

Criterion domain	Inclusion rule	Exclusion rule	Screening stage
Section A. PICOS eligibility criteria			
Population (P)	Core: adolescents/youth/students who are cyberbullying bystanders (witnesses of cyberbullying against a peer)	Workplace or general adult samples; no identifiable bystander/witness role. Seven retained boundary reports are separated from the core inference	Title/abstract + full text + sensitivity audit
Phenomenon of interest (I)	Factors (individual, relational, situational, sociocultural, platform) shaping bystander intervention/response online	Studies not addressing bystander intervention/response (perpetration- or victimization-only with no bystander outcome)	Title/abstract + full text
Context (C)	Cyberbullying/cyberaggression online; 2018–2026; peer-reviewed empirical	Online hate-speech-only; offline/traditional bullying only; non-empirical	Title/abstract + full text
Study designs (S)	Quantitative, qualitative, mixed-methods, experimental, RCT, psychometric/scale-validation	Reviews/meta-analyses; theses/dissertations; preprints/non-peer-reviewed	Title/abstract + full text
Time and source limits	2018–2026; peer-reviewed journal articles (databases or citation searching)	Pre-2018; non-bystander focus; non-peer-reviewed sources	Title/abstract + full text
Section B. Full-text exclusion reason codes (with counts)			
Code	Exclusion reason	*n* excluded	Provenance
EX1	Review/meta-analysis (not primary empirical)	3	File-verified (Domínguez-Hernández et al., 2018; Lo Cricchio et al., 2020; Machackova, 2020)
EX2	Offline/traditional bullying or peer aggression (not online CB)	1	File-verified (Mulvey et al., 2019)
EX3	Preprint/not peer-reviewed	1	file-verified (Chang et al., 2024)
EX4	Outside 2018–2026 window (pre-2018/seminal → background)	3	File-verified (Olenik-Shemesh et al., 2017; Patterson et al., 2017; Barlińska et al., 2013)
EX5	Residual group (no bystander-specific outcome, i.e., perpetration- or victimization-only; wrong adult/non-student population; online-hate-speech only; non-empirical/commentary)	148	Assessed at full text but not individually tallied by reason; grouped into one transparently named residual category (see Limitations)
**–**	**TOTAL full-text reports excluded (database arm)**	**156**	= 197 assessed − 41 included
EX-OM	Other-methods reports excluded	NR	The eight other-methods reports are an inclusion-stage count; the total candidate denominator and exclusions were not contemporaneously recorded

### 2.5 Study selection

**Figure 1:**
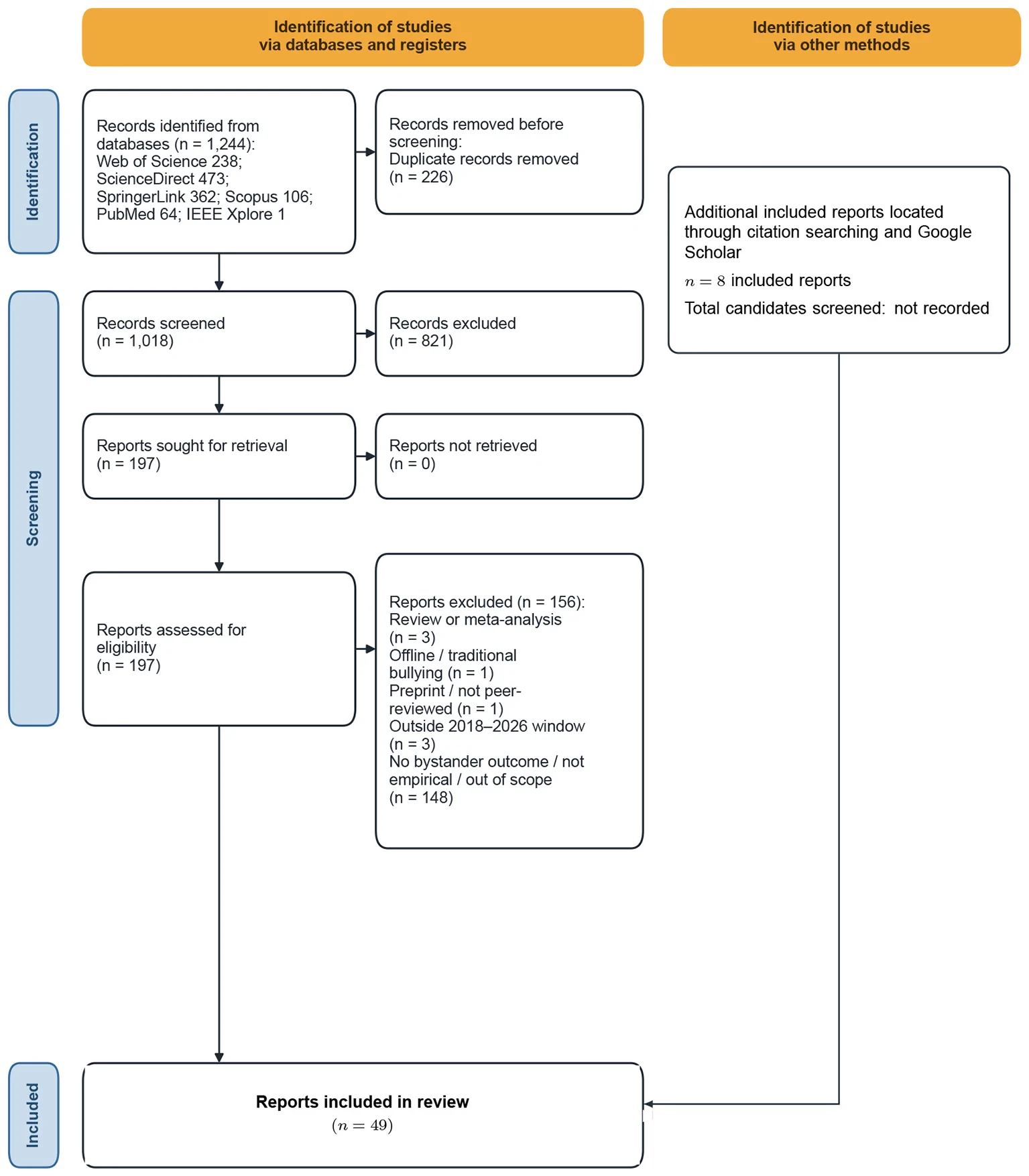
PRISMA 2020 flow diagram. The database arm yielded 41 reports. Eight additional included reports were located through citation searching and Google Scholar; the total number of candidates screened through those supplementary methods was not recorded, so the figure does not imply an eight-of-eight retention rate.

The selection process is depicted in the PRISMA 2020 flow diagram (Figure 1). The database searches identified 1,244 records (Web of Science 238, ScienceDirect 473, SpringerLink 362, Scopus 106, PubMed 64, IEEE Xplore 1). After 226 duplicates were removed, 1,018 unique records were screened on title and abstract, and 821 were excluded as clearly irrelevant. The remaining 197 reports were assessed at full text, and 156 were excluded against the codebook in Table 3. The largest group comprised reports that addressed no bystander-specific outcome, sampled the wrong population, concerned online hate speech only, or were non-empirical. Because those residual exclusions were not individually tallied by reason, they are grouped into one transparently labelled category and this limitation is revisited below. The database arm yielded 41 reports. Citation searching and Google Scholar located eight additional included reports, yielding 49 reports in the descriptive corpus. Importantly, eight is the number of included reports attributed to these supplementary methods, not the total number of Google Scholar or citation-search candidates screened. That candidate denominator and report-level source attribution were not preserved in the contemporaneous log; the revised flow diagram therefore labels them as not recorded rather than implying that eight of eight candidates passed every filter. Two reviewers screened independently and resolved disagreements by discussion; decision-support screening was used only to prioritize presentation order and did not exclude any record automatically. The included reports are listed with their coded attributes in Table 4.

**Table 4:** Characteristics of the 49 included studies.

References	Design	*N*	Tier	Domain	Quality
Arató et al. (2026)	Experiment	314	B	1,3,5	Some concern
Balakrishnan and Fernandez (2018)	Cross-sectional	1,263	A	1,2	High concern
Barlińska et al. (2013)	Experiment	271 (S1)/265 (S2)	A	1	Some concern
Barton et al. (2025)	Cross-sectional	257	A	1	Some concern
Chan et al. (2023)	Experiment	150	A	1	Some concern
Chen and Wu (2024)	Cross-sectional	1,716	A	1,4	Some concern
Civita et al. (2026)	Cross-sectional	90	C	3,4	Some concern
Clark and Bussey (2020)	Cross-sectional	540	A	1	Some concern
Feijóo et al. (2025)	Cross-sectional	225	A	1	High concern
García-Vázquez et al. (2024)	Cross-sectional	900	A	1,4	Some concern
Guo et al. (2025)	Experiment	271 and 196 (two online experiments; Weibo users)	B	3,6	Some concern
Herry and Mulvey (2023))	Cross-sectional	373	B	2,3	Some concern
Hu et al. (2023)	Cross-sectional	919 surveyed; 812 valid	A	1	Some concern
Huang et al. (2023)	Cross-sectional	189	A	2,3,4	High concern
Ireland et al. (2020)	Cross-sectional	316	B	1,3	Some concern
Li et al. (2024)	Cross-sectional	848	A	1,4	Some concern
Liu et al. (2021)	Cross-sectional	150 (Study 1; 2-study paper)	B	3	Some concern
Macaulay et al. (2020)	Cross-sectional	868	B	2,3	Some concern
Machackova et al. (2018)	Cross-sectional	443	A	1	Some concern
Nagar et al. (2022)	Experiment	119 (Canada 60; Iran 59)	C	3,5	Some concern
Schultze-Krumbholz et al. (2019)	Cross-sectional	726	B	1,4	Some concern
Sela-Shayovitz et al. (2024)	Cross-sectional	501	A	1	Some concern
Song and Oh (2018)	Cross-sectional	1,058	A	1,3	High concern
Tong and Talwar (2020)	Cross-sectional	166	B	1,4	High concern
Valdés-Cuervo et al. (2021)	Cross-sectional	1,674 (272 classrooms)	A	1	Some concern
Vlaanderen et al. (2020)	Cluster RCT	298	B	6	Some concern
Wang (2020)	Experiment	132	B	6	Some concern
Wang and Kim (2021)	Cross-sectional	2,888	A	1,2	Some concern
Xu and Han (2026)	Cross-sectional	1,563	A	1	Some concern
You and Lee (2019)	Experiment	253 (online experiment; adults, 138 anonymous condition)	B	3,6	Some concern
Zeng et al. (2023)	Experiment	247 (S1)/522 (S2)	B	2,3	Some concern
Zhao et al. (2023)	Cross-sectional	1,149	A	1,3	Some concern
Zhou et al. (2024)	Cross-sectional	1,952	A	1,4	Some concern
Hamzah et al. (2025)	Qualitative	32 (qualitative; 4 FGD sites)	A	1,6	High concern
Chen et al. (2025)	Cross-sectional	826	A	1,3	Low-to-some concern
Beavon et al. (2024)	Cross-sectional	839	A	3	Some concern
Chen (2024)	Cross-sectional	1,716	B	2,4	Some concern
Alcántar-Nieblas et al. (2024)	Psychometric	1,224 (612 + 612)	A	1	Low-to-some concern
Chen et al. (2024)	Cross-sectional	1,207	A	2,4	High concern
Gomes et al. (2024)	Mixed methods	140	C	3	Some concern
Jenkins et al. (2023)	Cross-sectional	872	A	2	Some concern
Biernesser et al. (2023)	Qualitative	46	B	6	Low concern
Luo and Bussey (2022)	Cross-sectional	563	A	1	Some concern
Fabris et al. (2022)	Cross-sectional	1,158	C	1	Some concern
Wright and Wachs (2022)	Longitudinal	1,067	C	1,3	Some concern
Hong and Lee (2022)	Cross-sectional	566	C	1	High concern
Doumas and Midgett (2020)	Cross-sectional	130	C	1,3	Some-to-high concern
Bussey et al. (2020)	Cross-sectional	540	A	1	Some concern
DeSmet et al. (2018)	Cluster RCT	216 (8th graders, two schools; 120 intervention/96 control analyzed)	A	6	High concern

Full coding matrix (country, verbatim design, age, platform, scenario, key factors, bystander outcome, main finding, citations) is provided as Supplementary Table S2. Outcome tiers: A, enacted or self-reported behavior; B, intention or willingness; C, affective or appraisal-related reactions. Domain codes: 1, individual psychological resources; 2, demographic and social-position; 3, incident appraisal and situational cues; 4, relational/family/school ecology; 5, sociocultural and normative; 6, platform affordances and intervention design. NR, not reported (qualitative study without an enumerated sample).

### 2.6 Data extraction and coding

For each included study, two reviewers extracted a common set of items into a structured charting form, reconciling discrepancies by discussion: country, design, sample size and composition, participant age, platform context, the bystander outcome examined, the predictors investigated, and the principal findings with the direction of association where available. The bystander outcome was coded along a behavioral spectrum (defending or active intervention; helping or emotional support; reporting or help-seeking; passive non-intervention; reinforcing or joining) so that studies measuring different points on the continuum could be compared on a common scale. Each outcome was further classified into one of three tiers: Tier A, enacted or self-reported behavior; Tier B, behavioral intention, willingness, or expected response; and Tier C, affective or appraisal-related reactions such as witnessing distress. Tier membership is recorded in Table 4. Predictors were organized with a six-domain coding scheme. (1) Individual psychological resources comprised affective and cognitive empathy, defender self-efficacy, moral identity, moral emotions, moral judgment, self-control, and self-esteem. (2) Demographic and social-position characteristics covered gender, age or school level, peer status, prior victimization, race or ethnicity, and social capital. (3) Incident appraisal and situational cues included perceived severity, urgency, perceived safety, victim self-disclosure, anonymity, and audience size. (4) Relational, family, and school ecology encompassed parental warmth and discipline, family emotional expressiveness, teacher support, school climate, and friendship quality. (5) Sociocultural and normative influences captured cross-national variation, social norms and expectations, and moral disengagement as a form of normative neutralization. (6) Platform affordances and intervention design spanned medium affordances, digital literacy and citizenship, and structured interventions such as serious games and near-peer mentorship. Studies could carry multiple tags, so the domain tallies reported in Section 3 exceed the number of studies. This dual coding of outcomes and domains provided the basis for the synthesis described in Section 2.8.

During revision, each report was also assigned an evidence-unit identifier. Reports were collapsed to one cohort unit only when identical sample size and composition or explicit report information confirmed participant overlap; shared authorship or membership in the same research program alone was not treated as proof of duplicate participants. The main tables retain report-level transparency, while Section 3.4 presents core-only and cohort-collapsed sensitivity counts.

### 2.7 Quality appraisal

Methodological quality was appraised with the Mixed Methods Appraisal Tool (MMAT, 2018 version) (Hong et al., 2018), selected because the evidence base spans qualitative, quantitative, and mixed-methods designs and the MMAT applies one coherent framework across all of them. For each study, two screening questions were answered first, followed by five design-specific criteria appropriate to the category of the study. All 49 included studies were appraised, and every appraisal was conducted from the complete published full text obtained through institutional access. Appraisal was not used as an inclusion filter: no study was excluded on the basis of its appraisal result. Quality information instead weighted confidence narratively, giving higher-quality studies greater interpretive weight within each factor domain. The criterion-level appraisal is presented in Section 3 and Table 5.

**Table 5:** MMAT 2018 quality appraisal of all 49 included studies.

References	Design/MMAT category	Items met (*n*/5)	Overall
Biernesser et al. (2023)	Qualitative	5/5	Low concern
Alcántar-Nieblas et al. (2024)	Quantitative descriptive	4/5	Low-to-some concern
Chen et al. (2025)	Quantitative descriptive	4/5	Low-to-some concern
Barton et al. (2025)	Quantitative descriptive	4/5	Some concern
Chan et al. (2023)	Quantitative non-randomized	4/5	Some concern
Civita et al. (2026)	Quantitative non-randomized	4/5	Some concern
Guo et al. (2025)	Quantitative non-randomized	4/5	Some concern
Herry and Mulvey (2023))	Quantitative non-randomized	4/5	Some concern
Ireland et al. (2020)	Quantitative non-randomized	4/5	Some concern
Macaulay et al. (2020)	Quantitative non-randomized	4/5	Some concern
Nagar et al. (2022)	Quantitative non-randomized	4/5	Some concern
Sela-Shayovitz et al. (2024)	Quantitative descriptive	4/5	Some concern
Valdés-Cuervo et al. (2021)	Quantitative non-randomized	4/5	Some concern
Wang (2020)	Quantitative non-randomized	4/5	Some concern
Wang and Kim (2021)	Quantitative descriptive	4/5	Some concern
Xu and Han (2026)	Quantitative descriptive	4/5	Some concern
You and Lee (2019)	Quantitative non-randomized	4/5	Some concern
Bussey et al. (2020)	Quantitative descriptive	3/5	Some concern
Arató et al. (2026)	Quantitative non-randomized	3/5	Some concern
Barlińska et al. (2013)	Randomized controlled trial	3/5	Some concern
Beavon et al. (2024)	Quantitative non-randomized	3/5	Some concern
Chen and Wu (2024)	Quantitative descriptive	3/5	Some concern
Chen (2024)	Quantitative descriptive	3/5	Some concern
Clark and Bussey (2020)	Quantitative descriptive	3/5	Some concern
García-Vázquez et al. (2024)	Quantitative non-randomized	3/5	Some concern
Gomes et al. (2024)	Mixed methods	3/5	Some concern
Jenkins et al. (2023)	Quantitative descriptive	3/5	Some concern
Li et al. (2024)	Quantitative descriptive	3/5	Some concern
Liu et al. (2021)	Quantitative descriptive	3/5	Some concern
Luo and Bussey (2022)	Quantitative non-randomized	3/5	Some concern
Machackova et al. (2018)	Quantitative descriptive	3/5	Some concern
Schultze-Krumbholz et al. (2019)	Quantitative descriptive	3/5	Some concern
Vlaanderen et al. (2020)	Randomized controlled trial	3/5	Some concern
Wright and Wachs (2022)	Quantitative non-randomized	3/5	Some concern
Zeng et al. (2023)	Randomized controlled trial	3/5	Some concern
Zhao et al. (2023)	Quantitative non-randomized	3/5	Some concern
Zhou et al. (2024)	Quantitative descriptive	3/5	Some concern
Fabris et al. (2022)	Quantitative descriptive	2/5	Some concern
Hu et al. (2023)	Quantitative descriptive	2/5	Some concern
Doumas and Midgett (2020)	Quantitative non-randomized	3/5	Some-to-high concern
Balakrishnan and Fernandez (2018)	Quantitative descriptive	2/5	High concern
Feijóo et al. (2025)	Quantitative descriptive	2/5	High concern
Hamzah et al. (2025)	Qualitative	2/5	High concern
Chen et al. (2024)	Quantitative descriptive	1/5	High concern
Hong and Lee (2022)	Quantitative non-randomized	1/5	High concern
Huang et al. (2023)	Quantitative descriptive	1/5	High concern
Song and Oh (2018)	Quantitative descriptive	1/5	High concern
Tong and Talwar (2020)	Quantitative non-randomized	1/5	High concern
DeSmet et al. (2018)	Randomized controlled trial	0/5	High concern

All 49 included studies were appraised from the full text of the published report. Screening items (S1, S2) and criterion-level ratings are shown in Figure 3; the full grid is Supplementary Table S3.

### 2.8 Synthesis method

Given this heterogeneity, findings were integrated through a structured synthesis consistent with the SWiM guideline (Campbell et al., 2020) rather than meta-analysis. Pooling was judged inappropriate because the designs do not yield commensurable effect estimates, because ostensibly similar coefficients refer to different points on the defending-to-reinforcing spectrum, and because predictors were measured with different instruments. Studies were grouped first by predictor domain and then by outcome family; within each domain, reported associations were tabulated by direction and by consistency across studies, with the volume of evidence recorded and the MMAT appraisal used to temper confidence. The domain-level patterns were then mapped onto a staged account of bystander response, from exposure and recognition through incident appraisal, moral-emotional activation, and efficacy and norm calibration to action selection, which is developed in the Discussion. Associations are described in hedged, non-causal language throughout, and no pooled effect sizes are reported.

Formal certainty grading (GRADE or GRADE-CERQual) was not applied, because those tools presuppose quantitative effect estimates or a body of qualitative findings addressing a single review question. In its place, Table 6 uses a transparent rubric. A domain shows consistent evidence when most contributing reports (approximately two-thirds or more) describe associations in the same direction and several rest on designs stronger than a single cross-sectional survey. It is labeled mixed when the direction varies, substantial null or contrary findings are present, or the evidence rests heavily on a small number of vignette demonstrations of uncertain real-platform generalizability. It is labeled emerging when fewer than five reports contribute or intention rather than enacted behavior dominates. These labels index volume and directional concordance tempered by the appraisal, not a formal certainty rating.

**Table 6:** Factor-domain synthesis and intervention implications.

Process stage	Factor domain (*n*)	Key specific factors	Direction/consistency	Candidate component or implication	Remaining gap
Moral-emotional activation	1. Individual psychological resources (*n* = 31)	Empathy (affective & cognitive); self-efficacy/defender self-efficacy; moral identity; moral emotions (guilt, sympathy); moral judgment; self-control; self-esteem; emotion regulation	Positive/consistent	Candidate to test: empathy, defender self-efficacy, and moral-emotional skills in anti-cyberbullying curricula.	Mostly cross-sectional; few experiments isolate which resource is causally decisive
Recognition	2. Demographic & social-position (*n* = 9)	Gender; age/school level; peer status; victimization experience; race/ethnicity; social capital	Mixed/mixed	No universal demographic targeting is supported; locally test whether gender, peer status, or prior victimization affects intervention response.	Gender effects inconsistent; peer status untested for cyberbullying specifically
Appraisal	3. Incident appraisal & situational cues (*n* = 18)	Perceived severity; emergency/urgency perception; perceived safety; incident interpretation; victim self-disclosure; anonymity; number of bystanders/group size; privacy context; collective signals	Positive/mixed	Candidate to test: severity recognition, safe-response scripts, and strategies to counter diffusion of responsibility.	Vignette-based evidence; audience-size thresholds and real-platform generalizability uncertain
Efficacy-norm calibration	4. Relational/family/school ecology (*n* = 10)	Parental warmth/support; parental rejection-overprotection; parental discipline style; family emotional expressiveness; teacher support; school climate; friendship quality; relationship to victim/bully	Positive/mixed	Candidate to test: parent, teacher, and school-climate components adapted to local institutional conditions.	Mostly indirect, mediated, and cross-sectional; one contrary parental-warmth finding
Efficacy-norm calibration	5. Sociocultural & normative (*n* = 2)	Culture/cross-national context (e.g., Canada vs. Iran); social expectations and norms; moral disengagement (normative neutralization)	Mixed/emerging	Candidate to test through local co-design: culturally appropriate pro-defending norms and norm-based messaging.	Only two studies; norm mechanisms inferred rather than measured
Action	6. Platform affordances & intervention design (*n* = 7)	Platform/medium affordances; anonymity and audience design; digital literacy/digital citizenship; serious games; near-peer mentorship; combined school + online programming	Positive/emerging	Candidate design options requiring evaluation: bystander prompts, reporting affordances, serious games, and near-peer mentoring.	Few controlled trials; intention outcomes dominate; short follow-ups

The direction/consistency column applies the evidence-confidence heuristic defined in Section 2.8. The candidate-component entries track the direction/consistency column; the platform and sociocultural entries rest on the thinnest evidence. Associated bystander outcomes per domain are listed in Table 4 and Supplementary Table S2.

## 3 Results

### 3.1 Study selection

The database searches returned 1,244 records across the six sources (Web of Science 238; ScienceDirect 473; SpringerLink 362; Scopus 106; PubMed 64; IEEE Xplore 1). After removal of 226 duplicates, 1,018 records were screened on title and abstract and 821 were excluded. The remaining 197 reports were assessed in full, and 156 were excluded against the codebook in Table 3. The database arm yielded 41 reports. Eight additional included reports were located through citation searching and Google Scholar, giving a descriptive corpus of 49 reports published between 2018 and June 2026. The eight supplementary-method reports were an inclusion-stage count, not the total number screened; the latter was not contemporaneously recorded and is shown as not recorded in the revised flow diagram. The characteristics of all 49 reports are summarized in Table 4; youth-focused interpretation is anchored in the 42-report core corpus and checked against 40 core cohort units after the two confirmed overlapping cohort pairs were collapsed in Section 3.4.

### 3.2 Characteristics of included studies

**Figure 2:**
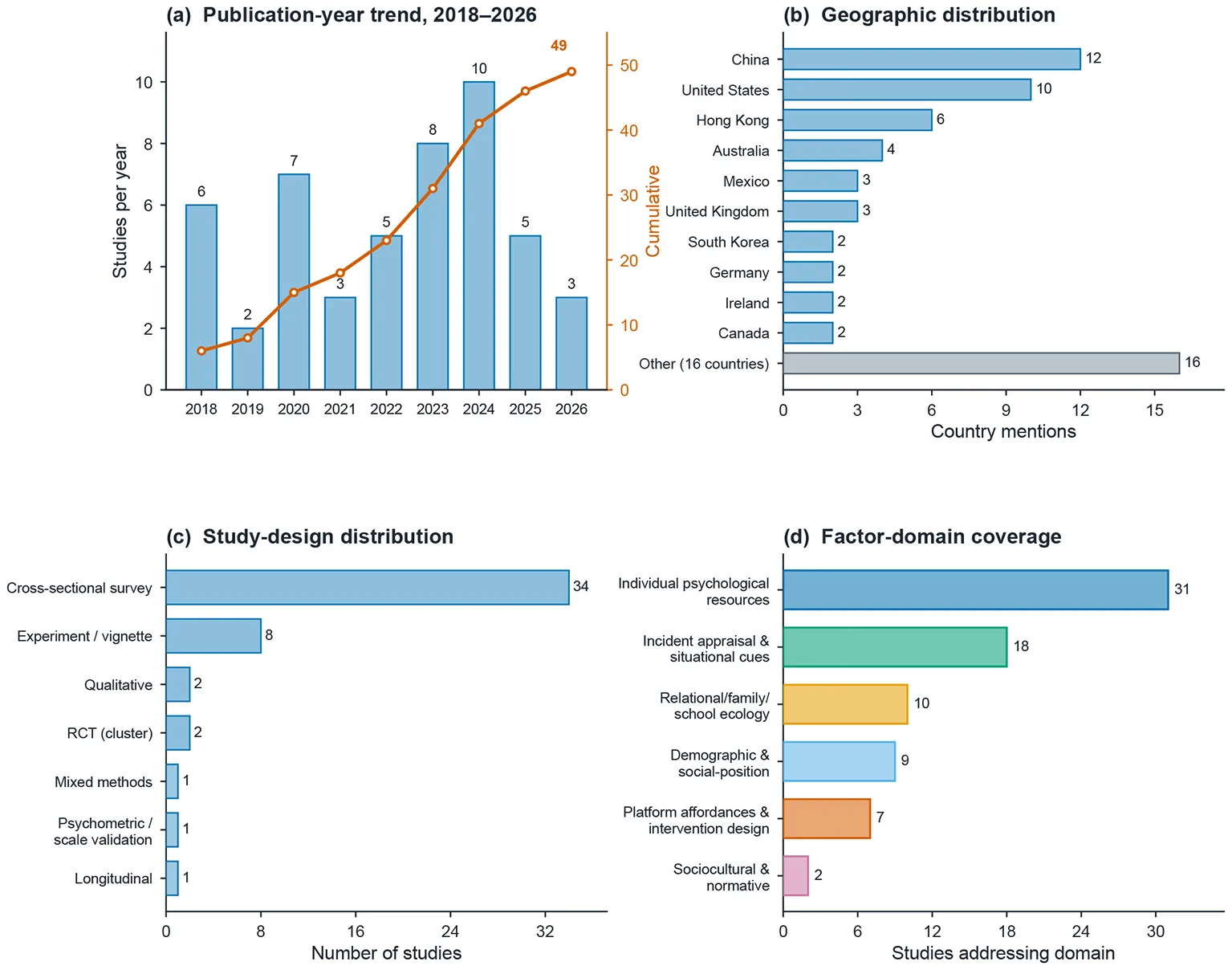
Evidence landscape of the 49 included studies: **(a)** publication-year trend, 2018–2026, with cumulative total; **(b)** geographic distribution (country mentions; multi-country studies contribute to each country); **(c)** study-design distribution; **(d)** factor-domain coverage. A study may address more than one factor domain, so the counts in **(d)** sum to more than 49.

The 49 included studies span the full 2018–2026 window, with output concentrated in the most recent years (Figure 2a). Annual output was modest in the early years (six studies in 2018, two in 2019, seven in 2020, three in 2021, and five in 2022), then rose sharply: eight in 2023, 10 in 2024, five in 2025, and three already captured for 2026 at the mid-year search date (Arató et al., 2026; Civita et al., 2026; Xu and Han, 2026). The earliest studies concentrated on individual psychological predictors, for example Song and Oh (2018) on behavioral reactions, Barlińska et al. (2013) and Machackova et al. (2018) on empathy, Balakrishnan and Fernandez (2018) on self-esteem, and DeSmet et al. (2018) on a serious-game intervention; later years progressively added situational, relational, normative, and platform-level considerations.

Geographically, the evidence concentrates in high-internet-penetration settings (Figure 2b). Mainland China contributed 12 studies (e.g., Chen et al., 2025; Hu et al., 2023; Li et al., 2024; Tong and Talwar, 2020; Xu and Han, 2026; Zhao et al., 2023; Zhou et al., 2024), the United States 10 (e.g., Biernesser et al., 2023; Doumas and Midgett, 2020; Herry and Mulvey, 2023; Ireland et al., 2020; Jenkins et al., 2023; Wright and Wachs, 2022; You and Lee, 2019), and Hong Kong six (e.g., Chen and Wu, 2024; Chen, 2024; Wang, 2020; Wang and Kim, 2021). Australia contributed four (Barton et al., 2025; Bussey et al., 2020; Clark and Bussey, 2020; Luo and Bussey, 2022); Mexico and the United Kingdom three each (Mexico: Alcántar-Nieblas et al., 2024; García-Vázquez et al., 2024; Valdés-Cuervo et al., 2021; United Kingdom: Macaulay et al., 2020 plus multi-country contributions); Canada two (Civita et al., 2026; Nagar et al., 2022); and South Korea, Germany, and Ireland two each (Hong and Lee, 2022; Song and Oh, 2018; Schultze-Krumbholz et al., 2019; Feijóo et al., 2025). Single-country contributions came from the Czech Republic (Machackova et al., 2018), Poland (Barlińska et al., 2013), Belgium (DeSmet et al., 2018), the Netherlands (Vlaanderen et al., 2020), Italy/Switzerland (Fabris et al., 2022), Israel (Sela-Shayovitz et al., 2024), Indonesia (Hamzah et al., 2025), Portugal (Gomes et al., 2024), Hungary (Arató et al., 2026), and Malaysia (Balakrishnan and Fernandez, 2018), alongside cross-national designs such as the Canada–Iran comparison in Nagar et al. (2022) and the multi-site samples of Feijóo et al. (2025), Sela-Shayovitz et al. (2024), and Wright and Wachs (2022). Overall, the bulk of the evidence originates from East Asia and North America, with the Global South sparsely represented.

The corpus was heavily weighted toward observational designs (Figure 2c). Thirty-four studies used cross-sectional surveys, frequently with mediation or moderated-mediation modeling (e.g., Chen et al., 2025; García-Vázquez et al., 2024; Hu et al., 2023; Xu and Han, 2026; Zhao et al., 2023; Zhou et al., 2024). Eight employed experimental or vignette manipulations: Barlińska et al. (2013) (empathy induction), You and Lee (2019) (anonymity × group size), Zeng et al. (2023) (self-disclosure and privacy), Wang (2020) (severity × interpersonal similarity), Nagar et al. (2022) (bystander type × relationship), Arató et al. (2026) (publicity, modality, and victim reaction), Chan et al. (2023) (a simulated-cyberbullying empathy experiment), and Guo et al. (2025) (two online experiments on victim disclosure and collective signals). The remainder comprised one cluster-randomized serious-game trial (DeSmet et al., 2018; *n* = 216 eighth-graders), one longitudinal study (Wright and Wachs, 2022), one psychometric scale validation (Alcántar-Nieblas et al., 2024), one school-randomized digital-citizenship intervention trial (Vlaanderen et al., 2020), two qualitative studies (Biernesser et al., 2023; Hamzah et al., 2025), and one mixed-methods serious-game study (Gomes et al., 2024). Report-level coverage across the six factor domains is summarized in Figure 2d. Adolescents in middle and high school predominated, with mean ages typically between 12 and 16 years (e.g., Hu et al., 2023, *M* = 12.70; Zhou et al., 2024, *M* = 14.18; Macaulay et al., 2020, 11–13 years; Chen et al., 2025, *M* = 15.82); a smaller set sampled university students or young adults, notably Balakrishnan and Fernandez (2018) (*M* = 20.9), Zeng et al. (2023) (*M* = 23.18 and 21.06), Xu and Han (2026), Zhao et al. (2023), and Herry and Mulvey (2023)) (*M* = 18.89). Samples ranged from small qualitative and experimental groups (e.g., 46 in Biernesser et al., 2023; 90 in Civita et al., 2026; 119 in Nagar et al., 2022; 132 in Wang, 2020) to school surveys exceeding 1,500 respondents (Chen and Wu, 2024; Chen et al., 2024; Hu et al., 2023; Xu and Han, 2026; Zhou et al., 2024) and one nationally representative survey of 2,888 adults (Wang and Kim, 2021). Most studies situated cyberbullying in general online or social-media contexts, with explicit social-media framing in You and Lee (2019), Wang (2020), Guo et al. (2025), and Chen et al. (2025). The outcomes examined spanned the full bystander spectrum, and several studies modeled constructive vs. aggressive defending as distinct outcomes (Alcántar-Nieblas et al., 2024; Barton et al., 2025; Bussey et al., 2020; García-Vázquez et al., 2024; Valdés-Cuervo et al., 2021). Classified by outcome tier, 28 studies measured enacted or self-reported behavior (Tier A), 14 measured intentions or willingness (Tier B), and seven measured affective or appraisal-related reactions (Tier C) (Table 4).

### 3.3 Methodological quality

**Figure 3:**
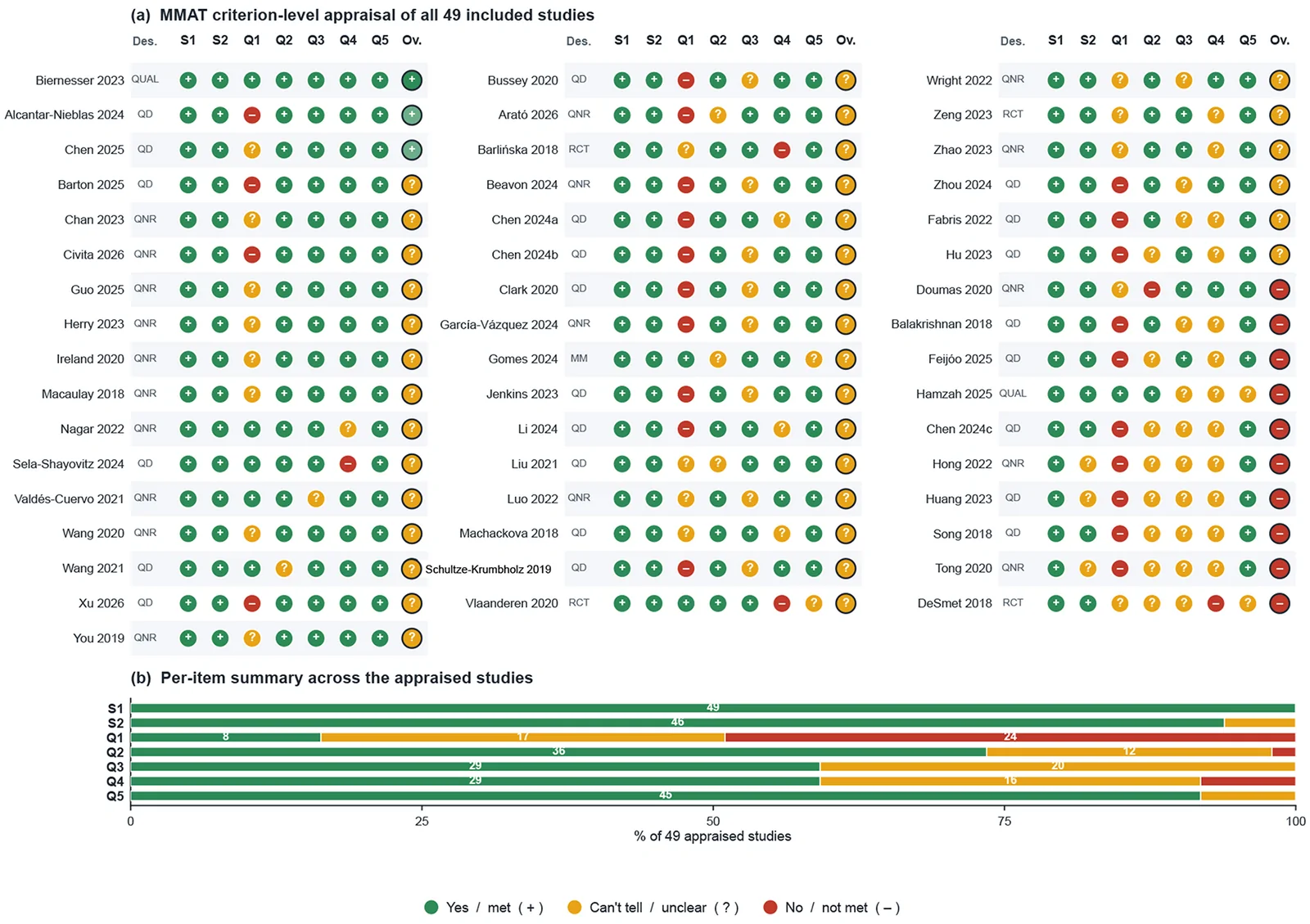
MMAT 2018 appraisal of all 49 included studies (traffic-light plot). S1–S2, screening items; Q1–Q5, design-specific quality criteria; Ov., overall rating; Des., MMAT design category (QD, quantitative descriptive; QNR, quantitative non-randomized; RCT, randomized controlled trial; MM, mixed methods; QUAL, qualitative). Green (+), criterion met; amber (?), cannot tell; red (–), not met. All 49 studies were appraised from the full text of the published report.

Methodological quality was appraised with the MMAT (2018). All 49 included studies were appraised, and every appraisal was scored from the full text of the complete published report, obtained through institutional access. The full set of criterion-level judgments is reported in Table 5 and displayed as a traffic-light plot in Figure 3. Twenty-three of the 49 studies were quantitative descriptive (e.g., Alcántar-Nieblas et al., 2024; Barton et al., 2025; Chen and Wu, 2024; Chen et al., 2025; Clark and Bussey, 2020; Jenkins et al., 2023; Li et al., 2024; Schultze-Krumbholz et al., 2019; Sela-Shayovitz et al., 2024; Wang and Kim, 2021; Xu and Han, 2026; Zhou et al., 2024), 19 were quantitative non-randomized (e.g., Arató et al., 2026; Civita et al., 2026; García-Vázquez et al., 2024; Guo et al., 2025; Herry and Mulvey, 2023; Hong and Lee, 2022; Ireland et al., 2020; Macaulay et al., 2020; Nagar et al., 2022; Valdés-Cuervo et al., 2021; Wang, 2020; You and Lee, 2019; Zhao et al., 2023), four were appraised against the randomized controlled-trial criteria (Barlińska et al., 2013; DeSmet et al., 2018; Vlaanderen et al., 2020; Zeng et al., 2023), two were qualitative (Biernesser et al., 2023; Hamzah et al., 2025), and one used mixed methods (Gomes et al., 2024).

Overall, the appraised evidence was of moderate quality: one report carried low concern, 38 some concern (a band including two rated low-to-some), and 10 high concern (a band including one rated some-to-high). The dominant weakness was sampling. Criterion Q1 was met in only eight of the 49 reports, while 24 received a “no” and 17 a “cannot tell,” because most surveys relied on convenience or single-region samples. Measurement was a second concern: the corpus is dominated by cross-sectional self-report surveys carrying common-method risk, and several reports used author-constructed or under-reported outcome instruments, including Chen (2024) and Fabris et al. (2022), and the partly validated measures in Doumas and Midgett (2020), Huang et al. (2023), and Tong and Talwar (2020). A cluster of reports nonetheless met four of five criteria, including the moderated-mediation survey (Chen et al., 2025), the serial-mediation survey (Xu and Han, 2026), the scale-validation studies (Alcántar-Nieblas et al., 2024; Sela-Shayovitz et al., 2024), the experimentally controlled designs (Ireland et al., 2020; Macaulay et al., 2020; Valdés-Cuervo et al., 2021; You and Lee, 2019), and the within-subjects vignette study (Civita et al., 2026). The strongest appraisal was the qualitative focus-group study (Biernesser et al., 2023), which met all five criteria; the weakest was the cluster-randomized trial (DeSmet et al., 2018), which met none, with randomization across only two schools and unestablished attrition. Five further reports fell into the high-concern band on one met criterion apiece (Chen et al., 2024; Hong and Lee, 2022; Huang et al., 2023; Song and Oh, 2018; Tong and Talwar, 2020). The appraisal therefore indicates a body of work whose internal validity is generally adequate but whose external validity and measurement rigor are uneven. Sensitivity to quality, scope, and non-independent cohorts is examined next.

### 3.4 Population-boundary and evidence-dependence sensitivity analyses

We conducted three linked checks (Table 7). First, removing the 10 high-concern reports did not reverse any domain-level direction/consistency label because those reports were distributed across domains. Second, removing the seven boundary reports defined in Section 2.4 reduced the descriptive corpus from 49 to 42 reports. The affected domain counts changed from 31 to 26 for individual psychological resources (Domain 1), 9 to 8 for demographic/social-position factors (Domain 2), 18 to 14 for incident appraisal (Domain 3), and 7 to 5 for platform/intervention factors (Domain 6); Domains 4 and 5 remained at 10 and 2. No domain-level direction/consistency label changed, and adolescent-focused statements in the Discussion are based on this 42-report core corpus.

**Table 7:** Sensitivity of factor-domain coverage to scope and confirmed cohort overlap.

Evidence set	D1	D2	D3	D4	D5	D6	Interpretation
All 49 reports	31	9	18	10	2	7	Descriptive map; includes seven boundary reports and two duplicate-cohort pairs
Core only (42 reports)	26	8	14	10	2	5	Youth-focused inferential base; all direction/consistency labels unchanged
Core, cohort-collapsed (40 units)	25	8	14	9	2	5	D1 remains positive/consistent; D4 remains positive/mixed

Counts are evidence reports in the first two rows and cohort units after confirmed overlap collapsing in the third. D1, individual psychological resources; D2, demographic/social position; D3, incident appraisal; D4, relational/family/school ecology; D5, sociocultural/normative; D6, platform/intervention design.

Third, we inspected report-level sample descriptions for overlap. Two pairs were confirmed to use identical cohorts. Clark and Bussey (2020) and Bussey et al. (2020) analyze the same Australian cohort of 540 students; they were therefore counted as one cohort unit in Domain 1. Chen and Wu (2024) and Chen (2024) report the same Hong Kong cohort of 1,716 adolescents with the same age distribution; they were counted as one cohort unit for coverage, reducing Domain 4 by one while retaining their distinct factor and outcome analyses descriptively. Other reports from the same research programs were not collapsed unless participant overlap could be confirmed. After collapsing the two confirmed overlapping cohort pairs, the core analysis comprised 40 cohort units: Domain 1 contained 25 cohort-collapsed core units and remained positive/consistent, while Domain 4 contained nine cohort-collapsed core units and remained positive/mixed. Thus, the report-level synthesis had overstated the volume of independent replication in these two domains by one unit each, but the correction did not alter the direction of either conclusion.

### 3.5 Factor-domain synthesis

RQ1 is addressed in Sections 3.5.1 to 3.5.6, which identify the factors examined and their directions of association; RQ2 is addressed by comparing the factor domains across the bystander outcome spectrum and tiers; and RQ3 is developed in Section 4.2 through the ecological-process model. The factors associated with bystander intervention were organized into the six domains defined in Section 2 and mapped to successive stages of the cyberbystander process (Table 6). The 49 reports generated 77 report-level domain tags, since most reports examined factors spanning more than one domain. Evidential weight differed markedly, from a large report-level body on individual psychological resources (*n* = 31; 25 cohort-collapsed core units in sensitivity analysis) to a very thin sociocultural literature (*n* = 2).

#### 3.5.1 Individual psychological resources

Individual psychological resources constituted the most studied and most internally consistent domain (31 of the 49 reports): greater empathy, stronger self-efficacy, and a more developed moral orientation were associated with more active and constructive responding. Empathy was the most frequently examined factor, with cross-sectional and experimental reports converging on a positive association with supportive responding (Chan et al., 2023; Hu et al., 2023; Li et al., 2024; Machackova et al., 2018; Schultze-Krumbholz et al., 2019; Wang, 2020; Zhao et al., 2023; Zhou et al., 2024). Several reports separated its components. Machackova et al. (2018) reported higher odds of supportive rather than passive responses at higher affective and cognitive empathy, Zhou et al. (2024) estimated a stronger indirect association for cognitive than affective empathy, and the experimental contrast in Barlińska et al. (2013) linked cognitive-empathy activation, but not affective-empathy activation, to more prosocial reporting. Chan et al. (2023) likewise observed more prosocial responses at higher levels of both components in a simulated incident. Defender self-efficacy was the second major resource: Clark and Bussey (2020) reported positive associations of defending and empathic self-efficacy with defending frequency, Song and Oh (2018) linked higher self-efficacy to more positive defensive behavior, and Ireland et al. (2020) associated individual and collective efficacy with greater willingness to intervene, with prosocial self-efficacy (Barton et al., 2025) and anti-cyberbullying self-efficacy (Feijóo et al., 2025) showing the same direction. Internet self-efficacy was modeled as a moderator of the empathy–helping association in Hu et al. (2023) and of the association between uncertainty stress and negative bystander behavior in Chen et al. (2025). Moral processes formed a third cluster. Xu and Han (2026) modeled belief in a just world and moral identity as serial mediators of the empathy–defending association, Hu et al. (2023) modeled internet moral judgment as a mediator between empathy and helping, Valdés-Cuervo et al. (2021) associated guilt and sympathy with more defending through self-regulation, and Hong and Lee (2022) identified five moral-emotion-defined bystander profiles. Conversely, Bussey et al. (2020) and Barton et al. (2025) linked moral disengagement to aggressive rather than constructive defending, and Luo and Bussey (2022) modeled it as a mediator between bystanding and later perpetration. Self-control (Sela-Shayovitz et al., 2024, in its low form linked to passive and pro-bully behavior) and self-esteem (Balakrishnan and Fernandez, 2018) completed the domain; the latter report found no significant empathy association with bystander action.

#### 3.5.2 Demographic and social-position factors

The demographic and social-position domain (nine studies) yielded a more mixed picture, with gender the most studied yet least consistent factor. Macaulay et al. (2020) found that positive responses rose with severity and that females showed a greater tendency to intervene, Wang and Kim (2021) reported in a nationally representative survey that women were more willing to intervene while men tended toward passivity, Herry and Mulvey (2023)) showed that women with prior experience of gender-based discrimination were more likely to expect to support victims, and Zeng et al. (2023) found female bystanders more likely to intervene in sexist situations. The effect was not universal: Barlińska et al. (2013) found no gender effect on prosocial reporting and García-Vázquez et al. (2024) reported no moderation of the modeled pathways, so gender effects were inconsistent across studies. Chen (2024) found a developmental shift in the form of responding, with middle-school students preferring to “ask for help” and high-school students more inclined to “call the police.” Peer status has been examined chiefly in the general school-bullying literature (Yang and Gao, 2023) and remains a gap for cyberbullying specifically. Prior cybervictimization showed bidirectional associations: positive with helping in Wang and Kim (2021) and Clark and Bussey (2020), but linked to fewer prosocial reactions in Chen (2024) and to negative bystander behavior via uncertainty stress in Chen et al. (2025). Chen et al. (2024) linked defending to greater offline social capital and academic achievement, while Jenkins et al. (2023) tested a bystander-intervention model across students of color and White students, underscoring that the process remains under-examined across racial and cultural groups.

#### 3.5.3 Incident appraisal and situational cues

The incident-appraisal domain (18 reports) indicated that how bystanders read an episode is associated with whether they act, although several key demonstrations come from vignette paradigms of uncertain real-platform generalizability. Perceived severity was the most consistently positive situational correlate: Macaulay et al. (2020) observed progressively more positive responses across severity levels, Ireland et al. (2020) associated willingness with incident interpretation and perceived severity, Huang et al. (2023) modeled an indirect association between severity and helping through sense of responsibility, and Zhao et al. (2023) modeled severity and empathy as serial mediators between prior experience and bystander behavior. Liu et al. (2021) associated emergency appraisal with greater empathy and felt responsibility. Victim cues also covaried with responses: Zeng et al. (2023) observed more intervention following positive victim self-disclosure in low-privacy settings and less following negative disclosure, while Guo et al. (2025) associated victim emotional disclosure and collective signals with stronger empathy and willingness to provide support. In an experiment, You and Lee (2019) observed a non-linear audience-size pattern in intervention intentions, with anonymity associated with direct but not passive intentions. Perceived safety emerged in Civita et al. (2026), where adolescents rated positive forms of support as safest. Relational appraisal featured in Nagar et al. (2022), where relationship to the perpetrator was associated with moral-responsibility attributions, and in Song and Oh (2018), where defending was more likely against a disliked bully. Arató et al. (2026) found that public, visual incidents with an evidently upset victim were judged most severe and interacted with individual characteristics, and Gomes et al. (2024) documented that worry and sadness were most prominent in the most serious scenario. Beavon et al. (2024) tested the five-step bystander-intervention model among 839 adolescents, finding sequential associations between adjacent steps, with prior bullying and victimization experiences related mainly to the earlier steps.

#### 3.5.4 Relational, family, and school ecology

The relational, family, and school ecology domain (10 reports) showed generally positive but predominantly indirect associations, typically modeled through empathy or efficacy. Li et al. (2024) associated parental warmth with defending through friendship quality and empathy as chain mediators, García-Vázquez et al. (2024) linked parental restorative discipline to constructive defending through justice sensitivity, and Zhou et al. (2024) modeled positive family emotional expressiveness as indirectly associated with more protective and less indifferent behavior through empathy. Chen and Wu (2024) supplied an instructive counter-current: teacher support and parental warmth were indirectly associated with positive bystander behavior through empathy, yet higher parental warmth was simultaneously associated with lower internet self-efficacy. Parental rejection and overprotection were associated with fewer prosocial reactions in Chen (2024), and parental online discipline style featured among the correlates in Huang et al. (2023). At the school level, Schultze-Krumbholz et al. (2019) associated school climate and empathy with intentions to assist or defend, and Chen and Wu (2024) identified a positive distal association for teacher support. Peer-relational quality appeared in Li et al. (2024) (friendship quality) and Chen et al. (2024) (offline social capital). Associations in this domain were almost entirely cross-sectional and mediated, with Chen and Wu (2024) providing the one contrary parental-warmth finding.

#### 3.5.5 Sociocultural and normative factors

The sociocultural and normative domain was the thinnest in the corpus (two studies) and should be regarded as emerging. Nagar et al. (2022) contrasted Canadian and Iranian youth and found that Canadian bystanders were more likely to condemn passive behavior and to support defending, whereas Iranian bystanders were more likely to endorse passivity. Significant interactions among country, the bystander's relationship to the perpetrator, and the response indicated that cultural context conditioned how relational cues were translated into moral judgments. Arató et al. (2026) positioned social expectations and moral disengagement, alongside empathy, as joint influences on whether cyberbystanders ignored, joined in, or intervened. Because moral disengagement functions in this domain as a normative neutralization mechanism, findings discussed under Domain 1 are conceptually relevant here as well, including its mediating role in Luo and Bussey (2022) and its association with aggressive defending in Bussey et al. (2020) and Barton et al. (2025). With only two studies directly targeting culture and social norms, cross-cultural moderation remains largely untested and the domain represents a clear evidence gap.

#### 3.5.6 Platform affordances and intervention design

The platform-affordances and intervention-design domain (seven reports) is best characterized as emerging, with most reports measuring intentions or program effects rather than observed defending. DeSmet et al. (2018) evaluated the Friendly Attac serious digital game in a cluster-randomized controlled trial of prosocial bystander behavior. In a school-randomized experiment, Vlaanderen et al. (2020) observed higher intervention intentions after a digital-citizenship program, although the hypothesized mediation through attitudes, norms, and perceived control was not supported. Gomes et al. (2024) used a pre/post serious-game design (“Com@Viver”) to examine witnessing emotions and regulation. The qualitative reports by Hamzah et al. (2025) and Biernesser et al. (2023) identified digital literacy as relevant to proactive anti-cyberbullying attitudes and a preference for near-peer mentorship with blended school-based and online programming, alongside hesitancy to report to parents or school staff. Platform affordances were examined in Wang (2020), where empathy and severity were associated with public and private intervention, and in You and Lee (2019), where anonymity and audience size were associated with direct-intervention intentions. Across these reports, bystander prompts, reporting affordances, digital-citizenship training, serious games, and near-peer mentorship were associated with more responding, but the evidence is limited by the scarcity of controlled trials, intention-dominated outcomes, short follow-ups, and the near-absence of platform-design effects tested at scale.

### 3.6 Evidence gaps and consistency across the corpus

Across the six domains, the corpus is uneven in both volume and consistency (Table 6). The most robust signal comes from Domain 1: empathy, defender self-efficacy, and a constructive moral orientation are positively associated with active responding across several design families. However, the sensitivity analysis reduces this domain from 31 report-level contributions to 25 cohort-collapsed core units, so the volume of replication is smaller than the unadjusted report count suggests. The affective-vs.-cognitive empathy distinction and occasional null findings (e.g., empathy in Balakrishnan and Fernandez, 2018) also leave the operative mechanism unsettled. Domain 3 is sizeable and directionally positive, but several key demonstrations rest on vignette and laboratory experiments (Arató et al., 2026; Wang, 2020; You and Lee, 2019; Zeng et al., 2023), so audience-size thresholds and real-platform generalizability remain uncertain. Domain 2 is mixed, with gender associations sometimes substantial and sometimes null. Domain 4 is positive/mixed but almost entirely cross-sectional and indirect; after confirmed cohort overlap is removed it contains nine cohort-collapsed core units rather than 10 reports. Domain 5 rests on only two reports, and Domain 6, despite containing two cluster-randomized trials (DeSmet et al., 2018; Vlaanderen et al., 2020), is dominated by intention outcomes and short follow-ups. Three cross-cutting limitations temper inference. Design: 34 of 49 reports are cross-sectional, only eight are experimental, just one is longitudinal, and only two are cluster-randomized, so temporal and causal claims are constrained and the intention–behavior gap unresolved. Geography: the evidence concentrates in China, the United States, and Hong Kong, limiting transferability. Measurement: bystander outcomes are operationalized heterogeneously and frequently rely on unvalidated self-report instruments, which impedes quantitative pooling and, together with the MMAT appraisal in Section 3.3, bounds confidence in the patterns above. The explicit dependence analysis in Section 3.4 prevents reports from confirmed identical cohorts from being interpreted as independent replications.

## 4 Discussion

### 4.1 Summary of main findings

Cyberbullying bystander intervention should be understood as an online social-dynamic process rather than only as an individual moral choice: whether a witness defends, supports, reports, stays passive, or reinforces is shaped jointly by the person, the relationships around them, the way the incident is read, and the affordances of the platform on which it unfolds. This review asked which factors shape bystander intervention in cyberbullying, how they cluster, and how they combine; the synthesis of 49 studies published between 2018 and 2026 suggests an answer to each question. For RQ1, the literature has examined a broad but recurring set of influences that distribute unevenly across six factor domains. Individual psychological resources are the most studied and most consistently positive domain (31 of the 49 studies), with empathy and self-efficacy repeatedly emerging as facilitators of defending and helping (Barlińska et al., 2013; Bussey et al., 2020; Clark and Bussey, 2020; Hu et al., 2023; Machackova et al., 2018; Valdés-Cuervo et al., 2021; Xu and Han, 2026). For RQ2, the remaining domains are better understood as conditional or contextual. Incident appraisal and situational cues were well supported but frequently design-qualified, with several of the most precise effects coming from vignette experiments (Huang et al., 2023; Ireland et al., 2020; Liu et al., 2021; Macaulay et al., 2020; You and Lee, 2019; Zeng et al., 2023). Relational, family, and school ecology (10 studies) operated largely indirectly, channeled through empathy or efficacy (Chen and Wu, 2024; García-Vázquez et al., 2024; Li et al., 2024; Schultze-Krumbholz et al., 2019; Zhou et al., 2024). Sociocultural and normative influences were examined in only two studies (Arató et al., 2026; Nagar et al., 2022), and platform affordances and intervention design were supported by seven studies that more often measured intention than behavior (Biernesser et al., 2023; DeSmet et al., 2018; Vlaanderen et al., 2020; Wang, 2020). Across domains, the bystander outcome spanned defending, support, reporting, passivity, and reinforcing, with different factors mapping onto different points of this spectrum. For RQ3, these patterns cohere when the factors are read not as parallel predictors but as inputs entering a staged, multilevel process, which the next section develops.

### 4.2 From factors to a staged ecological-process framework

**Figure 4:**
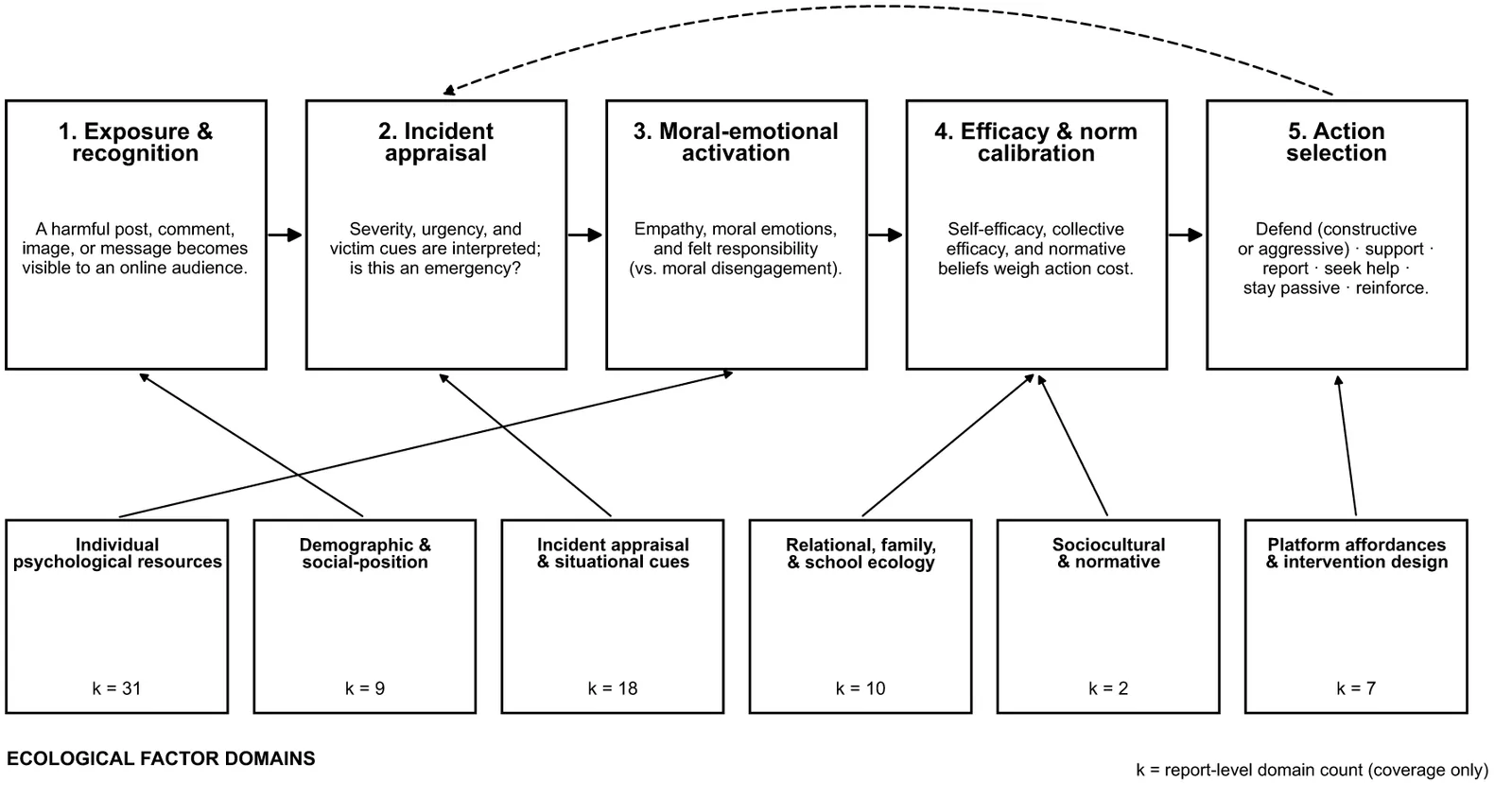
The cyberbystander ecological-process framework: a five-stage pathway from witnessing to action selection, shaped by six ecological factor domains. Connectors link each domain to the stage its factors most directly inform (a primary, not exclusive, locus); *k* is the report-level domain count and indexes coverage, not effect size, causal weight, or independent replication. Core-only and cohort-collapsed counts are reported in Table 7. The dashed arc denotes feedback, whereby observed outcomes inform subsequent appraisals.

**Figure 5:**
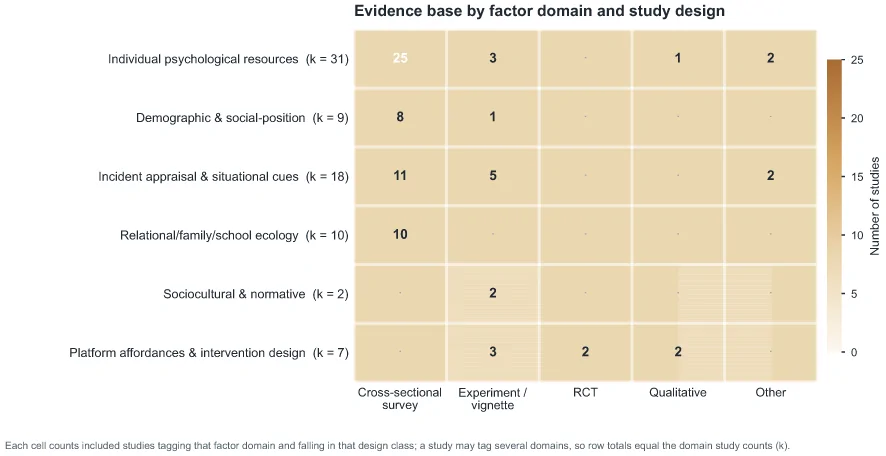
Evidence base by factor domain and study design: the number of included studies tagging each of the six factor domains within each design class. Because a study may tag several domains, row totals equal the domain study counts (*k*). Individual psychological resources and incident appraisal rest on the largest and most design-diverse bodies of work, whereas the sociocultural-normative and platform domains rest on few studies.

This review does not demonstrate a single causal sequence, and the staged structure it uses is not itself new: it builds on the bystander-decision tradition (Latané and Darley, 1970) and the ecological-systems perspective (Bronfenbrenner, 1979). The review's contribution is narrower and evidence-based: it organizes recurrent correlates into a heuristic five-stage framework of intervention readiness by placing each factor domain at the stage its factors most directly inform, and it makes explicit one stage, moral-emotional activation, that the classic decision model leaves implicit. Rather than competing, the domains can be read as entering at distinct points along a five-stage pathway: exposure and recognition, incident appraisal, moral-emotional activation, efficacy and norm calibration, and action selection. The pathway integrates a sequential decision logic (Latané and Darley, 1970) with the nested levels of a young person's social ecology (Bronfenbrenner, 1979) (Figure 4). Each factor domain is mapped to the stage its constituent factors most directly inform. These assignments mark a primary rather than exclusive locus, and demographic characteristics in particular plausibly moderate more than one stage. At exposure and recognition, demographic and social-position characteristics appear to shape noticing and the initial framing of what is seen (Chen et al., 2024; Herry and Mulvey, 2023; Jenkins et al., 2023; Macaulay et al., 2020; Wang and Kim, 2021) (with peer-status evidence drawn mainly from the general school-bullying bystander literature Yang and Gao, 2023). At incident appraisal, perceived severity and emergency raise the felt need to act, while anonymity, audience size, privacy conditions, and the visibility of victim distress shape whether the situation is read as requiring and permitting a response (Gomes et al., 2024; Huang et al., 2023; Ireland et al., 2020; Liu et al., 2021; Macaulay et al., 2020; You and Lee, 2019; Zeng et al., 2023). At moral-emotional activation, affective and cognitive empathy, moral emotions such as guilt and sympathy, and moral identity are associated with converting appraisal into a felt obligation to help (Barlińska et al., 2013; Hong and Lee, 2022; Hu et al., 2023; Machackova et al., 2018; Valdés-Cuervo et al., 2021; Xu and Han, 2026). At efficacy and norm calibration, individual self-efficacy operates alongside the relational ecology and sociocultural norms, which inform whether defending is expected or whether passivity is neutralized through moral disengagement (Arató et al., 2026; Bussey et al., 2020; Chen and Wu, 2024; Clark and Bussey, 2020; Luo and Bussey, 2022; Nagar et al., 2022; Quintero-Jurado et al., 2025; Schultze-Krumbholz et al., 2019; Zhou et al., 2024). At action selection, reporting routes, audience design, digital-citizenship competencies, and program-supplied response scripts shape whether readiness is expressed as defending, support, or reporting rather than dissipating into inaction (Biernesser et al., 2023; DeSmet et al., 2018; Hamzah et al., 2025; Vlaanderen et al., 2020; Wang, 2020; You and Lee, 2019). Read as a heuristic, the model helps reconcile apparent disagreements. Studies foregrounding empathy, school climate, or platform visibility illuminate different stages of one process. Empathy may be necessary for activation yet insufficient without efficacy or a safe response route, and effects estimated at one stage need not replicate where a different stage is the binding constraint. The framework also accommodates negative outcomes, since reinforcing can be understood as the action-selection endpoint when normative calibration favors the aggressor. The model should be interpreted as a heuristic integration of heterogeneous findings rather than an empirically verified causal sequence. Current evidence supports the relevance of appraisal, empathy, self-efficacy, and contextual support, but only a small number of studies directly test temporal or mediational links across these stages, so the model is best viewed as a framework for hypothesis generation and intervention design. The distribution of study designs across the six factor domains is summarized in Figure 5.

### 4.3 Bystander decision-making in a networked audience

The staged reading developed above is, at its core, a decision-making account, and reading the corpus through the classical psychology of helping clarifies why online settings are distinctive. In the situational model of helping, a witness must notice the event, interpret it as one that calls for help, assume responsibility, decide on a form of help, and act, with failure at any step ending the path to intervention (Latané and Darley, 1970). Each step is reshaped by the networked context. Noticing competes with a dense and continuous stream of content; interpretation is complicated by absent paralinguistic cues and by genuine uncertainty about whether a post is harmful or merely banter (Arató et al., 2026; Liu et al., 2021); and the assumption of responsibility is diluted across a large and often invisible audience, the online analogue of bystander apathy that You and Lee (2019) demonstrated experimentally when intervention intentions declined as the imagined audience grew. What the reviewed evidence adds to this skeleton is the weight of moral emotion at the junction where appraisal becomes action: empathy, guilt, and sympathy recur as the affective engine that converts a recognized incident into a felt obligation to help (Hong and Lee, 2022; Machackova et al., 2018; Valdés-Cuervo et al., 2021; Xu and Han, 2026), which is why the framework names moral-emotional activation as an explicit stage that the original decision model left implicit.

Locating these processes within cyberpsychology and the study of adolescent online behavior foregrounds a class of moderators with no clear offline equivalent: the affordances of the platform on which the incident unfolds. Anonymity can loosen accountability and shape whether a witness acts openly or only in private (Wang, 2020; You and Lee, 2019); audience size and the visibility of a victim's distress alter both the diffusion of responsibility and the empathic pull of the situation (Arató et al., 2026; Guo et al., 2025; You and Lee, 2019); and the persistence and shareability of harmful content mean that a single instance of non-intervention can be re-encountered and amplified long after the event. Design features intended to help, such as reporting buttons, comment controls, and the algorithmic prominence a post is given, operate as part of the decision environment rather than as neutral background, yet platform-level factors remain the most thinly studied domain in the corpus (DeSmet et al., 2018; Vlaanderen et al., 2020; Wang, 2020). Treating cyberbystander behavior as an online social-dynamic process rather than as a purely individual moral choice reframes these affordances as legitimate and modifiable targets for theory and for intervention, and it marks the point at which cyberpsychology can extend a literature that has so far concentrated on dispositional predictors. These findings also speak directly to educational psychology and school-based prevention, because adolescents' bystander responses are shaped within their peer, classroom, and family ecologies as much as by platform-mediated visibility, anonymity, audience effects, and reporting affordances, so prevention works best when school programs and platform conditions are addressed together.

### 4.4 Comparison with prior reviews

Situating these findings against earlier syntheses clarifies what this review adds (Table 1). The systematic review by Domínguez-Hernández et al. (2018) assembled 19 studies through 2016, predating the platform-era surge and offering no formal quality appraisal or cross-cultural coding; we extend coverage to 2026, synthesize 49 studies, apply the Mixed Methods Appraisal Tool, and move from a moderator list to a staged ecological-process account. Polanco-Levicán and Salvo-Garrido (2021) mapped the field on a base of nine articles with an emphasis on classifying bystander roles; this review targets instead the factors and stages that govern transitions along the outcome spectrum. Rudnicki et al. (2023) centered adults and online hate, leaving the adolescent, family, school, and platform ecology largely unaddressed; this review supplies that youth-focused, multilevel coverage while remaining specific to cyberbullying. Lo Cricchio et al. (2020) synthesized moral disengagement as a single mechanism across all involvement roles; here it is embedded within a broader factor set and located at the norm-calibration stage. Most directly, Jeyagobi et al. (2022) reviewed 27 studies published between 2012 and 2022 on the factors behind negative cyber-bystander behavior, without a formal quality appraisal and within a flat three-category scheme; the present review is complementary in both focus and method, covering the factors associated with intervention across the full defending-to-reinforcing spectrum, appraising all 49 included studies with the Mixed Methods Appraisal Tool, and organizing them into a staged ecological-process framework.

The staged structure itself is not novel. It descends from the situational decision model of helping (Latané and Darley, 1970), and several cyberbullying studies already treat responding as a multi-step process, whether through serious-game decision points (DeSmet et al., 2018) or sociocognitive models in which self-efficacy and moral disengagement gate defending (Barton et al., 2025; Bussey et al., 2020; Luo and Bussey, 2022). The contribution is a systematic mapping of factor domains to process stages. This mapping shows where the determinants literature is relatively dense (moral-emotional activation) and where it remains thin (norm calibration and platform design). It also shows that studies emphasizing empathy, school climate, or platform design address different parts of a longer proposed pathway.

Beyond period and scope, the review adds a transparent multi-database PRISMA search, MMAT appraisal, an explicit mapping of factor domains to process stages, and recent evidence from Chinese and other non-Western samples that earlier reviews could not capture (Chen et al., 2025; Hu et al., 2023; Li et al., 2024; Nagar et al., 2022; Zhou et al., 2024).

### 4.5 Practical and intervention implications

The staged model may inform a portfolio of complementary intervention components rather than a single universal program (Table 6). In adolescent school settings similar to those represented in the core corpus, empathy and moral-emotional exercises, defender-self-efficacy practice, and concrete response scripts are the best-supported candidate components (Barlińska et al., 2013; Clark and Bussey, 2020; Hu et al., 2023; Machackova et al., 2018; Valdés-Cuervo et al., 2021). Incident-appraisal activities may help students recognize severity and identify responses that are safe for both victim and witness (Civita et al., 2026; Ireland et al., 2020; Liu et al., 2021). Parent, teacher, and school-climate components are also plausible, but their evidence is predominantly cross-sectional and concentrated in Chinese, Hong Kong, United States, and selected European school contexts (Chen and Wu, 2024; Chen, 2024; Li et al., 2024; Quintero-Jurado et al., 2025; Schultze-Krumbholz et al., 2019; Zhou et al., 2024). These components should therefore be co-designed with local schools and families, checked against local reporting pathways and norms, and evaluated before wider transfer. The mixed gender and cultural findings do not support a universal assumption that one demographic group will respond in the same way across countries.

Platform-level proposals require still greater caution. Private support/reporting routes, bystander prompts, digital-citizenship training, serious games, and near-peer mentorship are testable design options rather than established cross-platform solutions (Biernesser et al., 2023; DeSmet et al., 2018; Hamzah et al., 2025; Vlaanderen et al., 2020; Wang, 2020). Much of their support comes from intention outcomes, small programs, or single-platform scenarios, and the review contains little evidence from low-connectivity settings, Africa, Latin America beyond Mexico, or South Asia. Accordingly, the school-based recommendations are most applicable to adolescent educational settings resembling the sampled contexts; family and normative components require cultural adaptation; and platform affordances should undergo local usability, safety, and effectiveness testing before deployment.

At the platform layer, friction prompts, private support or reporting routes, and visible pro-defending norms can be framed as a research agenda for the action-selection stage (Civita et al., 2026; Vlaanderen et al., 2020; Wang, 2020; You and Lee, 2019). The present corpus does not establish that these features work across platforms or jurisdictions. Any deployment should therefore be preceded by co-design with users, safeguarding review, and controlled evaluation of intended and unintended effects, including whether a feature exposes or burdens the victim. This cautious interpretation treats intervention as an online social process while recognizing that platform evidence is the thinnest domain in the review.

### 4.6 Limitations

Several limitations qualify these conclusions. First, 34 of the 49 reports are cross-sectional and self-reported, so most associations cannot establish temporal order or causation (Hu et al., 2023; Machackova et al., 2018). Many outcomes are intentions rather than observed behavior, leaving an intention–behavior gap. Second, most MMAT appraisals showed some concern, particularly for sampling representativeness and outcome measurement; a single cross-design tool is also less specific than design-focused instruments. Third, heterogeneity in design, population, predictor, and outcome precluded meta-analysis, so the synthesis addresses direction and coverage rather than pooled magnitude. Fourth, the original search used simple natural-language concept phrases, was restricted to English-language publications, and covered six sources; it may therefore have missed synonyms or records indexed elsewhere. The single original IEEE Xplore record illustrates this under-retrieval. A broader verification search on 30 July 2026 yielded 31 IEEE records, including six journal articles, but none of the six examined empirical determinants of human bystander intervention; this check did not add an eligible report, although it does not eliminate the possibility of omissions in other sources. Fifth, the evidence is concentrated in China, the United States, and Hong Kong. Practical suggestions are therefore most transferable to adolescent educational settings resembling those samples and should not be assumed to apply across cultures, platforms, or jurisdictions without adaptation and testing. Sixth, seven reports sit at the population, phenomenon, or outcome boundary. Youth-focused interpretation is anchored in the 42-report core analysis, not the full 49-report descriptive map. Seventh, after the two confirmed duplicate-cohort pairs were collapsed, the core analysis comprised 40 cohort units and the cohort-collapsed coverage in Domains 1 and 4 was reduced by one unit each; same-program studies with unconfirmed overlap may leave residual dependence. Finally, the supplementary-search candidate denominator and report-level source attribution were not contemporaneously retained: the eight other-methods reports are an inclusion count, not evidence that eight of eight Google Scholar or citation-search hits passed screening. Full-text exclusions beyond the named codebook categories were also aggregated, inter-rater agreement was not quantified, and reporting/publication bias could not be formally assessed. These audit-trail limitations reduce reproducibility and require the predominance of positive associations to be interpreted cautiously.

### 4.7 Future research

These limitations map onto a research agenda organized around the staged model. Most pressingly, longitudinal and experimental designs should test the stage transitions themselves, that is, whether appraisal precedes and predicts activation and whether efficacy and norm calibration moderate its conversion into action, allowing the model to be evaluated rather than only proposed (Clark and Bussey, 2020; Liu et al., 2021). Cross-cultural and platform-specific designs are needed to determine which stages are most binding in different normative and technological environments (Arató et al., 2026; DeSmet et al., 2018; Nagar et al., 2022). Because action selection is most often assessed by intention, validated measures of enacted bystander behavior would substantially strengthen the evidence on what converts readiness into defending, support, or reporting (Wang, 2020). The two thinnest domains, sociocultural norms and platform design, warrant targeted primary research, including controlled trials of platform affordances and norm-based interventions at scale (Biernesser et al., 2023; Vlaanderen et al., 2020). Where future studies report comparable effect metrics, a focused quantitative synthesis of the best-replicated pairs, such as empathy and defending, would also become feasible.

## 5 Conclusions

This review synthesized 49 empirical studies published between 2018 and 2026 to clarify the factors associated with bystander intervention in cyberbullying. Intervention is not driven by any single attribute; it emerges from the interplay of individual psychological resources, incident appraisal, the relational and institutional ecology, sociocultural norms, and platform affordances. Across the six domains, the evidence most strongly supports the roles of empathy, defender self-efficacy, and incident appraisal, whereas demographic, sociocultural, and platform-level influences remain mixed or comparatively under-studied.

These patterns motivate a staged, multilevel ecological model, spanning recognition, appraisal, moral-emotional activation, efficacy and norm calibration, and action selection, advanced as a testable proposal rather than a confirmed causal pathway. The model offers a way to organize a literature that has often appeared contradictory, on the hypothesis that factors which look decisive in isolation enter the process at different stages and depend on one another.

The practical implication is that prevention should move beyond urging young people to “be upstanders.” Programs are likely to be more effective when they build empathy and intervention self-efficacy, rehearse concrete and safe response scripts, clarify trustworthy reporting routes, and engage parents, teachers, peers, and platform design as mutually reinforcing supports. Realizing this agenda will require longitudinal and experimental tests of the proposed stage transitions, validated measures of enacted bystander behavior rather than intention alone, and greater attention to the currently thin sociocultural and platform-design evidences.

## Data Availability

The datasets and transparency materials supporting this study are available in the Open Science Framework at https://osf.io/89s3r. The review protocol is registered separately at https://doi.org/10.17605/OSF.IO/68CMH. Additional files are provided in Supplementary Tables S1–S4.

## Supplementary material

**Supplementary Table S1**

Exact original search queries and the dated IEEE verification search.

**Supplementary Table S2**

Study-characteristics, scope, and evidence-unit coding matrix.

**Supplementary Table S3**

Full MMAT 2018 appraisal grid.

**Supplementary Table S4**

The available 180-record documented screening subset.
